# The Development of Horns in Bovidae and the Genetic Mechanisms Underpinning This Process

**DOI:** 10.3390/biology14081027

**Published:** 2025-08-11

**Authors:** Xiaoli Xu, Wenwen Yan, Jiazhong Guo, Dinghui Dai, Li Li, Hongping Zhang

**Affiliations:** Farm Animal Genetic Resources Exploration Innovation Key Laboratory of Sichuan Province, College of Animal Science and Technology, Sichuan Agricultural University, Chengdu 611130, China

**Keywords:** bovine, sheep, goats, horn, morphology, genetic mechanisms

## Abstract

Horns in Bovidae, including bovines, sheep, and goats, are evolutionarily conserved cranial structures derived from cranial neural crest cells and composed of a bony core, dermis, epidermis, and keratinous sheath. Their development follows a shared trajectory across species, progressing through placode, fleshy, and mature stages. Genetic regulators such as *RXFP2*, *FOXL2*, *HOXD1*, and *TWIST1* have been identified as pivotal determinants controlling horn morphogenesis, sexual dimorphism, and the polled phenotype. This review synthesizes current advances in the evolutionary origins, morphological progression, and genetic regulation of horn formation in bovines, sheep, and goats to provide a comprehensive understanding of horn formation and variation. These findings lay the groundwork for future efforts to manipulate horn traits through genetic selection or genome editing, with implications for animal welfare and breeding.

## Simple Summary

Horns are unique cranial structures found in bovines, sheep, and goats, playing roles in defense, communication, and reproduction. Their development begins from neural crest cells and proceeds through conserved stages across species. Recent research has identified key genes and signaling pathways that regulate horn formation, shape, and the polled phenotype. Understanding the evolution, structure, and genetic control of horns helps explain their biological function and diversity and supports breeding efforts to reduce the need for dehorning, improving animal welfare in modern livestock systems.

## 1. Introduction

Ruminants are a diverse and successful group of herbivorous mammals characterized by their unique headgear and multi-chambered stomachs. This clade includes families such as Bovidae, Giraffidae, Cervidae, Rhinocerotidae, and Antilocapridae, with Bovidae alone comprising over 279 species [1]. Among ruminants, headgear morphology varies substantially: Cervidae have solid, cyclically shed and regrown antlers; Bovidae have enduring hollow horns; and Antelopes have pronghorns, which are an intermediate stage between solid and hollow [2]. These diverse horn types represent adaptive responses to varied ecological niches and selective pressures.

The evolutionary origins of horns in bovines, sheep, and goats trace back to the early and middle Miocene epochs, where ecological challenges and sexual selection shaped their emergence and diversification [3,4]. Historically, horns have served as indicators of breed quality and genetic fitness, influencing livestock breeding and management strategies. Nonetheless, horn presence introduces challenges, including increased feed requirements, elevated risk of injury during handling, and augmented aggression within herds [5]. Traditional disbudding methods, such as heat cautery and caustic paste application, although widely used, raise significant animal welfare concerns due to the associated pain and tissue damage. Consequently, there is growing interest in developing humane alternatives, including selective breeding for hornlessness [6]. These underscore the importance of elucidating the structural and genetic basis of horn development.

Horns originate from cranial neural crest cells and undergo a tightly regulated differentiation program that results in the formation of a complex organ composed of a bony core, periosteum, dermis, epidermis, and keratinous sheath, each essential for horn integrity and function [2]. Despite interspecific variation in horn size and morphology among bovines, sheep, and goats, their developmental progression is conserved, following sequential stages encompassing the horny placode, fleshy, and mature phases [7,8]. Crucial genetic loci and candidate genes implicated in horn development and morphology have been identified, including *RXFP2* [9], *FOXL2* [10], *MTX2* [11], *HOXD1* [12], *TWIST1* [13], and *ZEB2* [14,15]. Although previous research has characterized the genetic architecture of polledness in Bovidae, focusing primarily on the structural variants affecting these genes [5], relatively little attention has been devoted to the cellular and histological dynamics of horn morphogenesis. This review integrates insights from horn tissue architecture, morphogenetic progression, and genetic regulation to provide a comprehensive framework for understanding the complexity and diversity of horn development in ruminants.

## 2. Evolution and Functional Diversification of Horns in Bovidae

Horns are distinctive anatomical structures in domestic ruminants, playing multifaceted roles in ecological adaptation, intra-species communication, and cultural symbolism. These cranial appendages originate from a common ancestral trait and predominantly arise from cranial neural crest cells, which contribute to the embryonic facial mesenchyme rather than to the lateral and posterior cranial skeleton [2,16]. The diversification of horn morphology and structure among ruminants was largely shaped during the early to middle Miocene, driven by ecological pressures and the need for niche adaptation, resource competition, and sexual selection [17,18,19]. Horn evolution has also been hypothesized as a metabolic adaptation to seasonal environmental fluctuations, promoting phenotypic radiation in body size, behavior, and headgear morphology [20,21].

Functionally, horns serve as effective weapons in intra-species competition, offering an energetically efficient mechanism of combat via the development of a durable bony core [22]. In addition, horns may facilitate thermoregulation through specialized vascular structures that assist in heat dissipation [23]. In domesticated contexts, horns have historically been considered markers of breed quality and have influenced livestock selection based on horn shape and size. Culturally, horns hold symbolic and utilitarian value, being used in ornaments, musical instruments, and tools.

Despite their adaptive and cultural significance, the presence of horns poses practical challenges in modern livestock management. These include increased risks of injury to handlers and conspecifics, elevated feeding and space requirements, and greater animal aggression [5]. Consequently, breeding hornless cattle and goats has become a key goal in agricultural production. However, the emergence of polled intersex syndrome (PIS) in goats complicates selective breeding efforts. Female goats homozygous for the PIS locus exhibit intersexuality with associated reproductive tract malformations and infertility, while male goats of the same genotype remain phenotypically unaffected [24,25,26]. In practice, horn removal through electric dehorning or cauterization is widely employed to control horn development [27], yet these procedures raise ethical concerns due to the pain and tissue damage inflicted, conflicting with modern animal welfare standards.

## 3. Structure of Horns in Bovidae

**Figure 1:**
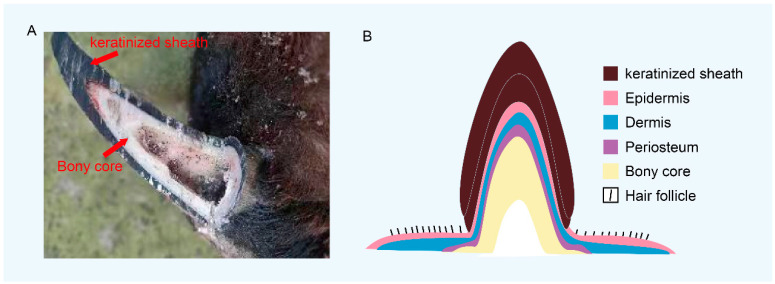
The structure of the hollow horns. (**A**) Anatomical illustration of goat horn structures. (**B**) Schematic diagram of the structure of the hollow horns.

Horns in Bovidae develop bilaterally from the frontal bones and are distinguished by their hardness, permanence, and structural complexity. Horn initiation begins with the induction of connective tissue and dermal components above the periosteum of the frontal bones, where horn buds emerge from a specialized connective tissue membrane [28]. As development progresses, fusion occurs between the frontal bones, the horn core, and the surrounding periosteum, forming a permanent, non-shedding cranial appendage, distinct from cervid antlers, which arise from pedicles and regenerate annually from periosteal outgrowths [29]. The mature horn is a composite structure derived from both mesodermal and ectodermal lineages, comprising a central bony core, periosteum, dermis, epidermis, and an outer keratinized sheath [28,30] (Figure 1). The horn core is vascularized and innervated, containing papillae, blood vessels, and nerve fibers, and is encased by a dense sheath of keratin that provides mechanical protection and rigidity [28,31]. Horn growth can occur through thickening around the base or radial expansion at the tip, reflecting species-specific growth dynamics. A notable anatomical adaptation in many bovid species is the pneumatization of the horn core, wherein osteoclast-mediated bone resorption creates internal cavities. This reduction in bone mass decreases the overall weight of the horn, thereby minimizing mechanical strain on the head and cervical spine [13]. Moreover, interspecific variation exists in the ultrastructure of horn tissues, including differences in the number and orientation of keratin tubules, lamellar organization, and keratinocyte morphology [32], contributing to the diversity of horn phenotypes observed among species.

## 4. Horn Morphological Development in Bovidae

Horn development in Bovidae species, mainly including bovines, sheep, and goats, shares notable similarities due to their conserved structural features and embryonic origins. These horns are specialized skin appendages consisting of a bony core covered by a keratinous sheath, the latter being secreted by differentiated epidermal keratinocytes [31]. Across species, horn morphogenesis proceeds through a series of conserved stages: early, middle, and late placode phases, followed by fleshy and mature stages. These stages are defined by the sequential development of epidermal thickening, hair follicle formation, dermal papillae proliferation, and progressive keratinization. In the early placode phase, basal cells are loosely arranged, and hair follicles are absent. As development advances to the middle stage, placodes form and are marked by elongated, spindle-shaped basal cells. During the fleshy stage, dermal papillae become more prominent, and the initial keratinous sheath begins to develop. By the mature stage, the horn is characterized by fully formed keratin layers and degenerated hair follicles integrated into the cornified structure [7,8]. Importantly, no bony core is observed during the embryonic stage, indicating that ossification is primarily a postnatal event [31,33].

In bovines, horn development begins as early as 58 days of gestation, with visible horn buds and localized depressions in horned but not in polled fetuses [34,35]. By day 70, histological differentiation of the horn bud becomes evident, and by day 115 in Holstein fetuses, dense nerve bundles emerge, suggesting a neurogenic contribution to horn development. Between days 155 and 268, increased epidermal keratinization and the appearance of skin appendages such as follicles and glands mark the transition to a mature horn bud [31]. In yak fetuses, similar concave horn bud structures with vacuolated cells are observed at 80–90 days [36].

Sheep horn development begins around day 70 of gestation, initially signaled by epidermal thickening [33,34]. In Altay sheep, horn buds display a significantly thicker epidermis at 105 days compared to the adjacent skin [37]. In Merino sheep, sexual dimorphism becomes apparent during fetal development: male fetuses exhibit horn bud formation as early as 75 days, while females lack observable buds from 55 to 84 days. At 84~88 days, the horn bud epidermis in males thickens to approximately 105 μm, compared to 75 μm in females, and dermal papillae begin to emerge. By day 118, the epidermis reaches ~260 μm and shows extensive vascularization. Postnatally, horn development continues with the thickening of both the epidermis and keratin sheath and the onset of underlying bone formation [33].

In goats, horn placode formation is first detected at 51 days of gestation, typically marked by the appearance of a small white dot on the frontal region [8]. Recent histological comparisons of the horn bud and forehead skin at embryonic days 50, 60, and 70 reveal that while no structural differences are observed at 50 days, the horn bud epidermis becomes significantly thicker than the forehead skin by 60 and 70 days. Moreover, dense mesenchymal fiber bundles, likely representing early nerve bundles, are specifically present beneath the horn bud dermis [38], indicating early regional specialization. Partial keratinization is observed around 21 days after birth, with complete keratinization and ossification by approximately 180 days postpartum [8]. Demographic studies indicate that goats reach 93% of their total horn length by three years of age. Sexual dimorphism is also present, with males exhibiting longer horns than females as early as 1.5 years [39]. However, compared to bovines and sheep, detailed histological and molecular characterization of horn development in goats remains limited, warranting further investigation.

## 5. Genetic Regulation of Horns in Bovidae

Horned is inherited as an autosomal recessive trait (p), while polledness is dominant (P). Mating between a horned individual (pp) and a homozygous polled (PP) will result exclusively in heterozygous polled offspring (Pp). In contrast, crosses between horned and heterozygous polled individuals can yield both polled and horned phenotypes. Notably, scurs, which are small, loosely attached horn-like growths, occur more frequently in heterozygous polled males, particularly when they carry one or two copies of the Sc allele [10,40]. Horns in Bovidae display extensive variation in morphology, size, and presence across species, reflecting distinct genetic regulatory mechanisms. In particular, horn development and polledness traits in bovines, sheep, and goats have been associated with multiple genomic loci and candidate genes (Table 1).

### 5.1. Genetic Regulation of Horns in Bovines

#### 5.1.1. Genes Governing Bovine Horn Development

The development of horns, as specialized derivatives of the skin, involves a complex interplay of genetic factors that regulate differentiation and maturation. Key genes, including *OLIG2*, *FOXL2*, and *RXFP2*, play crucial roles in neural crest cell differentiation, which is critical for the formation of large nerve bundles in fetal cattle horn buds [31,41]. Notably, the *RXFP2* gene is central to horn development, influencing both the initiation and growth of the horn structure [10]. Comparative proteomic analysis of horn buds from horned and polled yaks at embryonic days 80~90 revealed 29 upregulated and 71 downregulated proteins related to cell adhesion, motility, and metabolic activities [36]. In yak horn morphogenesis, Sonic Hedgehog (Shh) regulates differentiation within the basal epidermal layer, while β-catenin is involved in promoting cell proliferation, with significant expression in the hair follicle epidermis [42,43]. Understanding the genes, proteins, and molecular pathways involved in horn bud formation provides essential insights into the genetic basis of horn development.

#### 5.1.2. Genetic Regions and Genes Related to Bovine Polledness

Horn phenotypes in bovine species are typically categorized into normal horns, scurs, and polledness. Several genetic loci related to these traits are located on *Bos taurus* autosome 1 (BTA 1), a key region implicated in horn development [44,45]. A 0.8~2.8 Mb segment on BTA 1 contains genes such as *OLIG1*, *URB1*, *OLIG2*, *IFNAR1*, *C1H21orf62*, and *GART*, which have been linked to horn formation and polledness [41]. Four major P alleles of *Bos taurus* origin have been identified, each with distinct structural variants that contribute to the polled phenotype: (i) the Celtic PC allele involves the duplication of approximately 202 bp at the polled locus between the *IFNAR2* and *OLIG1* genes [46]; (ii) the Frisian PF allele is characterized by an 80 kb duplication, located 200 kb downstream of the PC allele [47]; (iii) the Mongolian PM allele involves a 219 bp complex duplication starting at 1,976,128 bp on BTA 1, along with a 7 bp deletion and 6 bp insertion located 621 bp upstream [48]; and (iv) the Nellore cattle harbor a novel 110 kb duplication variant at the polled locus [49]. Additionally, a 147 kb region encompassing *C1H21orf62*, *GCFC1*, and *SYNJ1* has been identified as the potential location of the POLL variant in yaks [50]. Interestingly, a long intergenic non-coding RNA (lincRNA#1) located 80 kb downstream of the PC allele is highly expressed in the horn buds of polled cattle, potentially inhibiting horn development. CRISPR/Cas9 experiments suggest that the deletion of a 3.7 kb lincRNA#1 segment or a 10 bp sequence at the polled locus alone is insufficient to induce a horned phenotype [46,47,51,52]. Polledness has been introduced into cattle populations through both traditional breeding and modern genome editing. For example, a genome-edited polled Holstein bull was produced via TALEN-based somatic cell nuclear transfer, successfully transmitting the trait to its offspring without off-target effects [53,54]. Additionally, CRISPR/Cas12a-mediated knock-in of the Celtic allele has resulted in hornless dairy cattle with stable inheritance [55]. In yak, naturally polled Ashidan yaks have been developed through long-term selective breeding, demonstrating the feasibility of conventional approaches in species adapted to high-altitude environments [56].

Beyond these loci, recent studies have highlighted the significant role of the *ZEB2* gene in polledness development. A 3.7 Mb deletion encompassing *ZEB2* has been linked to a novel polled and multisystemic syndrome that affects not only horn development but also facial dysmorphism and growth delay [15]. Furthermore, a de novo frameshift mutation in *ZEB2* in Fleckvieh cattle, which results in a truncated protein and symptoms similar to human Mowat–Wilson syndrome, has been reported, leading to developmental delays, small stature, and cranial abnormalities [14].

#### 5.1.3. Genetic Regions and Genes Linked to Bovine Scurs

Scurs are an autosomal recessive trait, presenting as smaller, unstable, horn-like structures loosely attached to the skull. The genetic basis of scurs is complex, with key loci mapped to BTA 19, located 4 cM distal to BMS2142 and 6 cM proximal to IDVGA46, with *ALOX12* and *MFAP4* being the nearest genes [57]. Genetic studies of scurs in polled cattle suggest a polygenic basis, with multiple loci involved in modulating the phenotype [58]. A genome-wide association study (GWAS) conducted in Simmental cattle uncovered an association between scurs and BTA 19, underscoring the role of this locus in scur development [59]. The manifestation of scurs is further modulated by sex and genotype, with the PF allele demonstrating a more pronounced effect in mitigating scur development [58].

Scurs can also result from mutations in other loci. One example is *TWIST1* on BTA 4, where a frameshift mutation in exon 1 is linked to type 2 scurs in French Charolais cattle [13]. This mutation is associated with scurs in heterozygous individuals, while homozygous mutations are embryonically lethal, highlighting the complex genetic architecture underlying this trait.

### 5.2. Genetic Regulation of Horns in Sheep

#### 5.2.1. Genes Related to Horn Development in Sheep

Horn development in sheep is influenced by a complex interplay of genes involved in neurogenesis and osteogenesis. Genes such as *SNAI1*, *SNAI2*, *SOX10*, and *TFAP2A*, related to neural development, were highly expressed in the horn bud at embryonic day 90 of sheep, according to RNA-seq analysis [30]. Furthermore, RNA-seq studies comparing horn buds and skin at embryonic day 105 in Altay sheep revealed 68 differentially expressed genes (DEGs), including *RXFP2*, *FOXL2*, and *TNN*, which are potentially involved in regulating horn development through the WNT signaling pathway [37]. *RXFP2*, a gene that encodes a G protein-coupled receptor, plays a crucial role in horn traits in sheep [9]. *RXFP2*-deficient mice exhibit a reduction in bone mass, mineralizing surface, bone formation rate, and osteoclast surface area, indicative of compromised skeletal integrity and function [60]. Additionally, proteomic analysis of horn tissues from Altay sheep, representing different phenotypes such as scurred, regular, and four-horned, identified 232 differentially expressed proteins (DEPs) associated with the scur phenotype. Key pathways, including the EMC–receptor interaction pathway, focal adhesion pathway, and PI3K-Akt signaling pathway, were found to be significantly linked to several DEPs, including THBS1, COL6A2, COL6A3, COL6A1, COL1A2, ITGAV, FN1, TNC, and TNN. These proteins are implicated in the normal development and differentiation of the horn structure in sheep [61]. These findings suggest that the mechanisms governing horn development in bovine and sheep species may share common molecular pathways, involving genes like *RXFP2* and *FOXL2*, indicating potential evolutionary conservation.

#### 5.2.2. Genetic Regions and Genes Controlling Sheep Polledness

Sheep horns exhibit wide variability in size, shape, and number, categorized into polledness, normal, and polycerate phenotypes. The genetic locus responsible for polledness in sheep is localized to a region of approximately 7.4 cM on chromosome 10 (OAR 10) [62]. The polled phenotype is primarily caused by the insertion of a 1833 bp segment at the 3′ end of the *RXFP2* gene, within a 4 kb region on OAR 10. This insertion introduces a potential antisense RNA sequence of the eukaryotic translation elongation factor *EEF1A1*, which effectively blocks the transcription of *RXFP2* [9]. Other genes, including *OarHH41*, *AGLA226*, and *EEF1DP3*, are also believed to influence the polledness phenotype in sheep [63,64,65].

#### 5.2.3. Genetic Regions and Genes Controlling Polycerate Sheep

The polycerate phenotype, characterized by the presence of multiple horns, is a distinct trait in sheep and is associated with a specific genetic locus located on OAR 2. This locus is marked by several strongly linked single-nucleotide polymorphisms (SNPs) within the 131.9~132.6 Mb region in Navajo-Churro and Jacob sheep [66], and in the 132.6~132.7 Mb region in Altay, Mongolian, and Sishui Fur sheep [67]. The genes closest to these associated markers are *MTX2* and the *HOXD* cluster, which plays a key role in limb and organ development [66,67]. Notably, the 132.9~133.1 Mb region on OAR 2 has been identified as a strong candidate genomic region for the polycerate phenotype in Sishui Fur and Damara sheep, encompassing seven HOXD genes (*HOXD1*, *HOXD3*, *HOXD8*, *HOXD9*, *HOXD10*, *HOXD12*, *HOXD13*) and *EVX2*, *MTX2*, *MIR10B*, and *KIAA1715* [68,69]. The polycerate phenotype in sheep and goats is directly linked to the dysregulation of *HOXD1*, a gene involved in transcriptional regulation during embryogenesis. In sheep, a 4 bp deletion in *HOXD1* disrupts splice donor sites, resulting in a truncated protein. This defect impairs transcriptional regulation, leading to the splitting of horn buds and the formation of multiple horns. Similarly, the polycerate phenotype in goats is associated with structural variations affecting *HOXD1*. A 137 kb insertion from chromosome 5 into chromosome 2, coupled with a 503 kb deletion at the insertion site, alters *HOXD1* expression patterns, impacting horn development and resulting in the polycerate phenotype [12]. These findings emphasize the conserved role of *HOXD1* in horn development across Bovidae, providing key insights into the genetic regulation and evolution of horn traits in ruminants.

### 5.3. Genetic Regulation of Horns in Goats

The polled phenotype in goats is governed by an autosomal dominant gene (P), which is expressed in both homozygous dominant (PP) and heterozygous (Pp) individuals, while homozygous recessive (pp) goats exhibit horns [70]. Interestingly, intersexuality is observed only in homozygous polled females, while homozygous polled males remain phenotypically normal and fertile [26]. Recent studies have identified an 11.7 kb deletion at approximately 129 Mb on chromosome 1 as a key factor in PIS in goats, disrupting the transcription of genes such as *FOXL2*, *PISRT1*, and several non-coding RNAs [24]. Further refinement of this deletion reveals a 10,159 bp region accompanied by a 480 kb duplication containing the genes *KCNJ15* and *ERG* [71,72,73]. Polymorphisms in *FOXL2* and *PISRT1* have been strongly linked to PIS in dairy goats [74,75], with *FOXL2* playing a critical role in ovarian development [76]. In Tangshan dairy goats, specific markers, including partial 11.7 kb deletions, 198 bp substitutions, and 108 bp deletions, have been identified, likely contributing to the intersex condition [77]. Notably, the genetic linkage map of chromosome 1 in goats reveals distinct features compared to bovines [78], indicating that the genetic mechanisms underlying polledness in goats involve a complex interplay of multiple factors [79].

**Table 1:** Genetic regions and candidate genes associated with hollow horns.

Species	Phenotypes	Positions	Candidate Genes	Ref.
Bovine	Polled	BTA 1: 0.8~2.8 Mb	*OLIG1*, *URB1*, *OLIG2*, *IFNAR1*, *C1H21orf62*, *GART*	[41]
		BTA 1: 147 kb	*C1H21orf62*, *GCFC1*, *SYNJ1*	[50]
		BTA 2: 3.7 Mb	*ZEB2*	[15]
	Scurs	BTA 19: BMS2142 and IDVGA46 regions	*ALOX12*, *MFAP4*	[57,80]
		BTA 4: 1.7 Mb	*TWIST1*	[13]
Sheep	Polled	OAR 10: 1833 bp	*RXFP2*	[9]
	Polycerate	OAR 2: 132.9~133.1 Mb	*MTX2*, *HOXD*, *EVX2*, *MIR10B*, *KIAA1715*	[68]
Goat	Polled	Chr 1: 11.7 kb	*FOXL2*, *PISRT1*	[24]
		Chr 1: 10.159 kb deletion and 480 kb duplication	*KCNJ15*, *ERG*	[71,72,73]

## 6. Environmental Influences on Horn Development

In addition to genetic regulation, horn development is influenced by environmental conditions, such as nutrient availability and temperature, which can affect the rate and extent of horn growth. Horn growth in bighorn sheep is influenced by resource availability, particularly during early life. When food is limited, younger males tend to allocate more energy to body growth than to horn development, potentially prioritizing survival over secondary sexual traits [81]. However, the relationship between resource availability and horn growth varies with age and body condition. Similarly, research on Alpine ibexes has shown that horn growth in males is positively influenced by spring temperatures, likely due to earlier vegetation onset and improved food availability during warmer springs [82]. Moreover, early life conditions can have effects on horn development and reproductive patterns. In chamois, females with greater early horn growth tended to reach primiparity at a younger age, indicating a link between early horn development and reproductive timing [83]. Similarly, in Japanese serow, horn growth records were closely associated with reproductive history, with horn increment patterns reflecting fertility rates and age at first reproduction [84]. Habitat characteristics, particularly geological substrate, can influence horn growth by shaping local ecological conditions such as food availability, snow cover, and slope aspect. These environmental features may affect access to nutrients or minerals essential for horn development [85], highlighting the importance of considering ecological context when interpreting the phenotypic variation.

## 7. Cellular Heterogeneity Underlying Horn Development

Horn development in Bovidae (bovines, sheep, and goats) is a complex process involving the coordination of mesodermal and ectodermal cell populations. These cells contribute to the formation of the bony core and keratinized sheath, which are essential for horn structure and function. Recent advances in single-cell RNA sequencing (scRNA-seq) have uncovered the cellular heterogeneity underlying horn development. In dairy goats, 10 distinct cell types have been identified, including dermal cells, endothelial cells, epithelial cells, hair follicle cells, nerve cells, horn-associated keratinocytes/stromal cells, immune cells, myoblasts, neural crest/chondrocytes, and peripheral cells. Each of these cell types contributes uniquely to horn morphogenesis through specific signaling pathways and differentiation programs. Among them, nerve cells play a pivotal role in initiating horn bud formation by regulating the differentiation of mesenchymal stem cells and epidermal keratinocytes. Neural crest/chondrocytes serve as a key population to form the bony core. Horn-associated keratinocytes contribute to the formation of the outer sheath, undergoing terminal differentiation and keratinization, while immune cells and endothelial cells may support tissue remodeling and angiogenesis during horn growth [38]. In Cervidae, scRNA-seq atlases constructed across various stages of antler regeneration (from 10 days before casting to 90 days after casting) have revealed that PRRX1+ mesenchymal cells dominate the early stages of regeneration. These cells play a crucial role in initiating tissue remodeling and driving the regenerative process [86]. Furthermore, the androgen–RXFP2 axis emerges as a key regulatory pathway in antler development, with RXFP2 acting as a marker for antler stem cell lineage [87,88]. These insights into cellular dynamics not only deepen our understanding of horn and antler biology but also provide possibilities for exploring genetic selection for hornless traits in livestock.

## 8. Conclusions

Recent advances in genomics and RNA-seq have deepened our understanding of horn development in Bovidae, revealing key genes like *RXFP2*, *FOXL2*, and *TWIST1* that regulate this trait. While progress has been made in identifying genetic pathways for horn formation, critical questions remain regarding the molecular mechanisms behind the skin-to-horn transformation and the genetic diversity of horn morphology across species. Comparative studies across bovines, sheep, and goats are essential to further unravel the evolutionary processes and refine genetic selection strategies. Future research should focus on functionally validating candidate genes through in vivo and in vitro models, with targeted genome editing (e.g., CRISPR/Cas9) to assess their roles. Single-cell and spatial transcriptomics can further resolve cellular heterogeneity and differentiation trajectories within the horn tissues. Integrating epigenomic tools such as ATAC-seq and ChIP-seq will help identify regulatory elements controlling horn ontogeny. Together, these strategies will provide mechanistic insights and enable the precise genetic engineering of hornless livestock, promoting both animal welfare and production efficiency.

## Data Availability

Data sharing is not applicable to this article.
